# Drought neutralises plant–soil feedback of two mesic grassland forbs

**DOI:** 10.1007/s00442-018-4082-x

**Published:** 2018-02-05

**Authors:** Ellen L. Fry, Giles N. Johnson, Amy L. Hall, W. James Pritchard, James M. Bullock, Richard D. Bardgett

**Affiliations:** 1 School of Earth and Environmental Sciences, Michael Smith Building, The University of Manchester, Oxford Road, Manchester, M13 9PT UK; 2 NERC Centre for Ecology and Hydrology, Maclean Building, Benson Lane, Crowmarsh Gifford, Wallingford, Oxfordshire OX10 8BB UK

**Keywords:** Plant–soil feedbacks, Soil, Drought, Resource capture, Plant functional traits

## Abstract

Plant–soil feedbacks (PSFs) describe the effect of a plant species on soil properties, which affect the performance of future generations. Here we test the hypothesis that drought alters PSFs by reducing plant–microbe associations and nutrient uptake. We chose two grassland forb species, previously shown to respond differently to soil conditioning and drought, to test our hypothesis. We conditioned unsterilised grassland soil with one generation of each species, and left a third soil unconditioned. We grew a second generation consisting of each combination of plant species, soil, and drought in a full factorial design, and measured soil microbial community and nutrient availability. *Scabiosa columbaria* displayed negative PSF (smaller plants) under non-droughted conditions, but neutral under drought, suggesting that drought disrupts plant–soil interactions and can advantage the plant. Photosynthetic efficiency of *S. columbaria* was reduced under drought, but recovered on rewetting regardless of soil conditioning, indicating that PSFs do not impede resilience of this species. *Sanguisorba minor* showed positive PSFs (larger plants), probably due to an increase in soil N in conspecific soil, but neutral PSF under drought. PSF neutralisation appeared to occur through drought-induced change in the soil microbial community for this species. When *S. minor* was planted in conspecific soil, photosynthetic efficiency declined to almost zero, with no recovery following rewetting. We attributed this to increased demand for water through higher demand for nutrients with positive PSF. Here we show that drought neutralises PSFs of two grassland forbs, which could have implications for plant communities under climate change.

**Electronic supplementary material:**

The online version of this article (10.1007/s00442-018-4082-x) contains supplementary material, which is available to authorized users.

## Introduction

Plant–soil feedbacks (PSFs) occur when a plant species changes the microbial, chemical, or physical properties of the soil it is grown in (termed soil conditioning), in a manner that affects the performance of subsequent generations of plants either positively or negatively (van der Putten et al. 2013). While there is extensive literature concerning the response of plants to soil conditioning, the interaction between PSFs and abiotic stress has been largely overlooked. Drought, in particular, is likely to impact plant performance both directly through water stress, and indirectly via either changes to the structure and function of the microbial community, or by preventing plants from accessing soil nutrients (Meisner et al. 2013; Kaisermann et al. 2017). Further, shifts in soil microbial community composition resulting from PSFs could influence the capacity of plants to tolerate drought in subsequent generations (Lau and Lennon 2011, 2012), although our knowledge of how microbial communities influence plant traits and plant responses to drought is limited (Friesen et al. 2011; Lau and Lennon 2011). Water relations impact the ability of soil microbial communities to function and interact with plant roots, and it has recently been shown that drought can have strong legacy effects on PSFs and plant competitive interactions via modification of microbial communities (Kaisermann et al. 2017). Therefore, it is likely that linkages between plant roots and microbial communities would become decoupled under drought, which could advantage plant species that are adversely affected by microbially driven PSFs, or disadvantage those that are favourably affected by PSFs.

Soil conditioning also modifies soil nutrient availability (Bezemer et al. 2006), which in turn impacts plant traits involved in resource capture, especially root branching and proliferation; roots may receive lower investment in non-structural carbohydrates when proliferation is rapid (Hodge 2004; Levang-Brilz and Biondini 2002). It is likely that the traits of individual species will vary according to both nutrient availability and drought, but the outcomes of these interacting effects are poorly understood. Increased nutrient availability has been shown to increase a plant’s demand for water, which could exacerbate drought effects. The paucity of knowledge regarding the effects of PSFs on the ability of plants to tolerate drought, and of the biotic and abiotic mechanisms involved, represents an important knowledge gap, given predicted increases in extreme climate events (Classen et al. 2015; Fry et al. 2016). As such, incorporating PSFs into studies of plant species responses to drought could improve understanding of complex non-linear effects of drought on ecosystem structure and functioning (Classen et al. 2015).

The objective of this study was to examine how drought modifies PSFs of two grassland forbs in terms of both their biomass and plant traits, and photosynthetic efficiency following drought and rewetting. Specifically we tested the hypotheses that: (1) drought decreases strength of PSF by reducing the strength of plant–microbe interactions, and reducing soil nutrient uptake rates; and (2) PSFs will directly affect a plant’s response to drought, through changes in biomass and plant functional traits. This was tested in a greenhouse experiment using two plant species, *Scabiosa columbaria* and *Sanguisorba minor*, which co-occur in species-rich calcareous grassland, and have similar average effect trait values (characteristics of plants that affect their environment, such as root area; Hill et al. 2004). Previous studies have shown that *S. columbaria* displays negative feedback, performing worse in its own soil, whereas *S. minor* displays positive feedback, in that it performs better in conspecific than heterospecific soil (Bezemer et al. 2006). Moreover, these two species are known to have different responses to drought: *S. minor* is able to retain leaf turgor under severe drought, which is thought to be a result of accessing water in deeper soil layers (Buckland et al. 1997), whereas *S. columbaria* wilts under drought, but displays high resilience to water loss and recovers turgor when conditions improve (Buckland et al. 1997; Stampfli and Zeiter 2004). Therefore, while these two species have similar ‘effects’ traits, one species shows negative PSFs, but tolerates drought through physiological control, while the other shows positive PSFs, but is a drought avoider, which may make individuals more vulnerable to drought if no water is detected in deeper layers. These contrasting strategies may point to contrasting responses when these species are subject to both drought and PSF.

To test our hypotheses, we carried out a mesocosm study consisting of a conditioning and subsequent feedback phase for both species, which enabled us to compare growth responses of each species when grown in both conspecific and heterospecific soil, with field soil as a baseline to identify changes brought about by the two test plant species. We further compared plant responses to drought when they had been grown in differently conditioned soil, which allowed us to test whether the effects observed were due to drought or a legacy of prior soil conditions. We measured photosynthetic efficiency (*F*_V_/*F*_M_) as a response trait to enable us to track the effect of drought on each species over time, as well as recovery upon rewetting. If there was a stronger effect of drought on conspecific soils than heterospecific conditioning, this would indicate that PSFs conferred a disadvantage. To identify the potential mechanisms that underpin observed effects, we also measured a series of effect traits, including root area, leaf and root nitrogen (N) content (LNC and RNC), specific root length (SRL) and specific leaf area (SLA), to determine whether the plant species had shifted their traits according to PSFs, and whether this had any effect on resistance to and recovery from severe drought. Using root traits to describe the effects of soil treatments is intuitive because they form the interface between plants and soil, and so changes to the soil microbial community or soil nutrient status will be closely linked to changes in root tissue chemistry and structure (Bardgett et al. 2014). Finally, we measured soil microbial community composition at both generation points and soil nutrient availability to inform on potential mechanisms that underpin observed changes in PSFs.

## Methods

### Experimental setup

Soil was collected from a cattle and sheep-grazed semi-natural chalk grassland on Salisbury Plain, Wiltshire, UK, September 2013 (lat: 51.105, long: 0.390). The grassland was last ploughed in the 1940s, and comprised a lowland calcareous grassland (CG3a) community according to the UK National Vegetation Classification (Rodwell 1992). The soil was a sandy loam type on a chalk substratum, averaging 53% sand and 6% clay (Soil Survey Staff 1999). Total soil C (accounting for carbonates) was 9.46%, while total soil N was 0.83%. Soil pH was 7.93. The soil was passed through a 2-mm sieve to remove stones and small roots, and stored in a cold room at 5 °C prior to establishing the experiment. Two co-existing forb species, *S. columbaria* and *S. minor*, were used in this experiment; these species have similar effect trait syndromes, and different abundances in the field (Table 1). The experiment consisted of two phases: a soil conditioning phase and a soil feedback phase. The feedback experiment was conducted using unsterilised field soil in order to reduce the undesirable effects of sterilising soil, including increased nutrient status and invasion of greenhouse microbial pests (Diez et al. 2010; Brinkman et al. 2010). It had the advantage of beginning with a natural microbial community and nutrient status, which could be differentially altered by the two plant species in the conditioning phase. A disadvantage of this approach is that changes in microbes or nutrients could be small and difficult to detect. Therefore, we compared two types of conditioned soil with the natural field soil (after van der Putten et al. 2007; Engelkes et al. 2008).

**Table 1:** Database-derived characteristics of the two experimental species

Species	Height (cm)^a^	Perenniality^b^	Root type^c^	Specific leaf area (mm^2^ mg^−1^)^b^	Mean seedbank density (1/m^2^)^b^	Field abundance (% cover)^d^
*S. columbaria*	70	Biennial–perennial	Tap	19.04	13	Low (~ 3%)
*S. minor*	50	Perennial	Tap	20.64	42	High (~ 15%)

^a^PLANTATT (Hill et al. 2004)

^b^Leda traitbase (Kleyer et al. 2008)

^c^Grime (Grime et al. 2007)

^d^Field data from species-rich calcareous grassland, (E.L. Fry, unpublished data)

For the conditioning phase, seeds were bought from Emorsgate Seeds (King’s Lynn, UK) and germinated on 1% agar in a growth chamber set to 20/10 °C day/night with a 12/12 h photoperiod. When the plants acquired four leaves after the cotyledons, they were transferred to a glasshouse with an ambient temperature of 20 °C and 12 h of lamp light per day. The soil was distributed evenly between nine pots consisting of three conditioning treatments: (1) an unplanted control; (2) conditioning with the *S. columbaria* seedlings; and (3) conditioning with *S. minor* seedlings (both ten seedlings per pot). Soils were conditioned by growing plants for 63 days, after which seedlings were carefully removed, soil brushed off and returned to the pots, and soil from each conditioning treatment was well mixed. A small sample of each soil was frozen at – 20 °C for microbial community analysis (see below).

For the feedback phase, the final full factorial design consisted of 3 soil-conditioning treatments × 2 plant species × 2 watering regimes × 5 replicates = 60 pots. These were fully randomised. Seeds from both species were germinated on agar as above and then grown until four leaves appeared. The soil from each conditioning treatment was evenly reallocated to 20 pots, and these were planted with a single seedling per pot and placed in trays in the growth chambers. We began the drought treatment when plants were 119 days old. Soil moisture characteristics had been calculated previously using equations of hydraulic properties based on the known particle sizes of the soil (Saxton et al. 1986). Specifically, well-watered pots were maintained at saturation point (~ 36% soil moisture content; SMC) and the drought pots at just above wilt point (~ 7% SMC). These watering treatments were maintained for 5 weeks through weighing and maintaining pots at individually calculated weights. After 5 weeks, all the pots were watered to raise SMC back to saturation point for 1 week. At this point the plants had been in the pots for 8 months. They were then harvested and soil was collected and carefully mixed and stored at 5 °C prior to analyses of nutrient availability and microbial community. A subset of soil was frozen at – 20 °C for phospholipid fatty acid (PLFA) analysis.

### Photosynthetic efficiency measurements

Every week before watering during the drought phase, soil moisture content (% SMC) was measured in the pots using a Thetaprobe with HH2 attachment (Delta-T Instruments, Cambridge, UK). To assess the drought stress of individual plants, we then measured chlorophyll fluorescence parameters of one healthy leaf of each plant with a FluorPen (FT100, Photon Systems Instruments, Drasov, Czech Republic). Plants were placed in the dark for 2 h, before measurement of maximum photosystem II quantum yield (*F*_V_/*F*_M_). The mean *F*_V_/*F*_M_ for healthy plants is 0.83 (Bjorkman and Demmig 1987; Johnson et al. 1993). Stressed plants have a lower *F*_V_/*F*_M_ reflecting light-induced oxidative damage to the photosystem II reaction centre (Maxwell and Johnson 2000). We measured *F*_V_/*F*_M_ every week for 5 weeks, with the penultimate week receiving the deluge rewetting, so we could assess recovery.

### Trait measurements

At the end of the feedback experiment, leaves from each plant were counted, and the aboveground biomass was harvested at the soil surface and leaves cut and scanned for surface area using a scanner and WinRhizo^®^ root analysis software (Regents Instruments, Canada). The leaves were then dried for 24 h at 80 °C and weighed, and SLA was calculated by dividing mass by area. Roots were carefully removed from the soil and washed. Surface area, root volume, length and diameter were then determined using WinRhizo. Roots were then dried for 24 h at 80 °C and weighed. Root-to-shoot biomass ratios were calculated. SRL was determined by dividing root area by root length, and both shoot and root C and N were determined using a dry combustion elemental analyser (Elementar vario EL cube, Hanau, Germany).

### Soil measures

Inorganic N in soil from the feedback phase (NH_4_ and NO_3_) was analysed using a 5 g subsample of soil extracted with 25 ml of milli-Q water and shaken for 10 min at 150 rpm (Allen 1989). Extracts were then passed through Whatman No. 1 filter paper and analysed colorimetrically on an autoanalyser (AA3 HR AutoAnalyser, Seal Analytical, Soton, UK).

Frozen soil was used for PLFA, using the method described by Frostegård et al. (1991) and Bardgett et al. (1996). Extracted fatty acid methyl esters were analysed on an Agilent Technologies 7890B gas chromatograph with an Agilent DB-5 ms column. For bacterial biomarkers, we used the fatty acids i15:0, a15:0, 15:0, i16:0, 17:0, i17:0, cy17:0, *cis*18:1ω7 and cy19:0, and for the fungal biomarker we used 18:2ω6 (Bardgett et al. 1996; De Vries et al. 2015).

### Statistical analysis

All statistical analyses were carried out using R 3.1.0 (R Core Team 2012). First, soil microbial community composition, after the conditioning phase and then after the feedback phase, was evaluated using non-metric multidimensional scaling (NMDS) with Bray–Curtis distance measures (vegan package of R; Borcard et al. 2011). Then total PLFA values, fungal PLFA an bacterial PLFA were tested for normality and transformed as appropriate, and responses to treatments were analysed using one-way ANOVA for the conditioning phase, using soil conditioning as the explanatory variable, and soil conditioning and drought and an interaction term as the explanatory variables after the feedback phase.

ANOVAs were carried out to test whether the three soil-conditioning treatments affected plant biomass and trait and soil N availability, and whether there was an effect of drought, with a two-way interaction term between soil conditioning and drought. Non-constancy of variances was evaluated using Levene’s test in the car package of R (Levene 1960), and normality was ascertained and corrected for where necessary using Box–Cox transformations in the MASS package (Box and Cox 1964). PSFs were calculated for individual replicate pots as follows: 100(*T*_conspecific_ − *T*_heterospecific_)/(*T*_heterospecific_), where *T*_heterospecific_ refers to average heterospecific biomass or trait values (conditioned with the other species), and *T*_conspecific_ refers to individual biomass or trait values for plants grown in conspecific (conditioned with the same species) soil (van der Putten et al. 2007; Baxendale et al. 2014). These feedbacks were calculated separately for droughted and well-watered so we could identify whether PSFs modified drought responses. By standardising the values against their respective conspecific average, we could test whether the soil-conditioning treatment exacerbated or dampened drought effects on plant traits and performance. *S. columbaria* and *S. minor* were evaluated separately, so we could compare relative feedbacks for each species in conspecific relative to heterospecific soils under both drought and well-watered conditions. These feedbacks were evaluated using a two-tailed *t* test to see if there was a deviation from zero.

Finally, we analysed the effect of soil conditioning on plant physiological stress using dark-adapted *F*_V_/*F*_M_ values. This was used as the response variable in repeated measures ANOVA that considered each species separately, with soil conditioning and drought as the explanatory variables with an interaction term, and an error structure that incorporated the time element. These models were not simplified. We followed this by testing for treatment effects on soil inorganic N availability using the same model structure as before.

## Results

### Phase one: soil conditioning

**Fig. 1:**
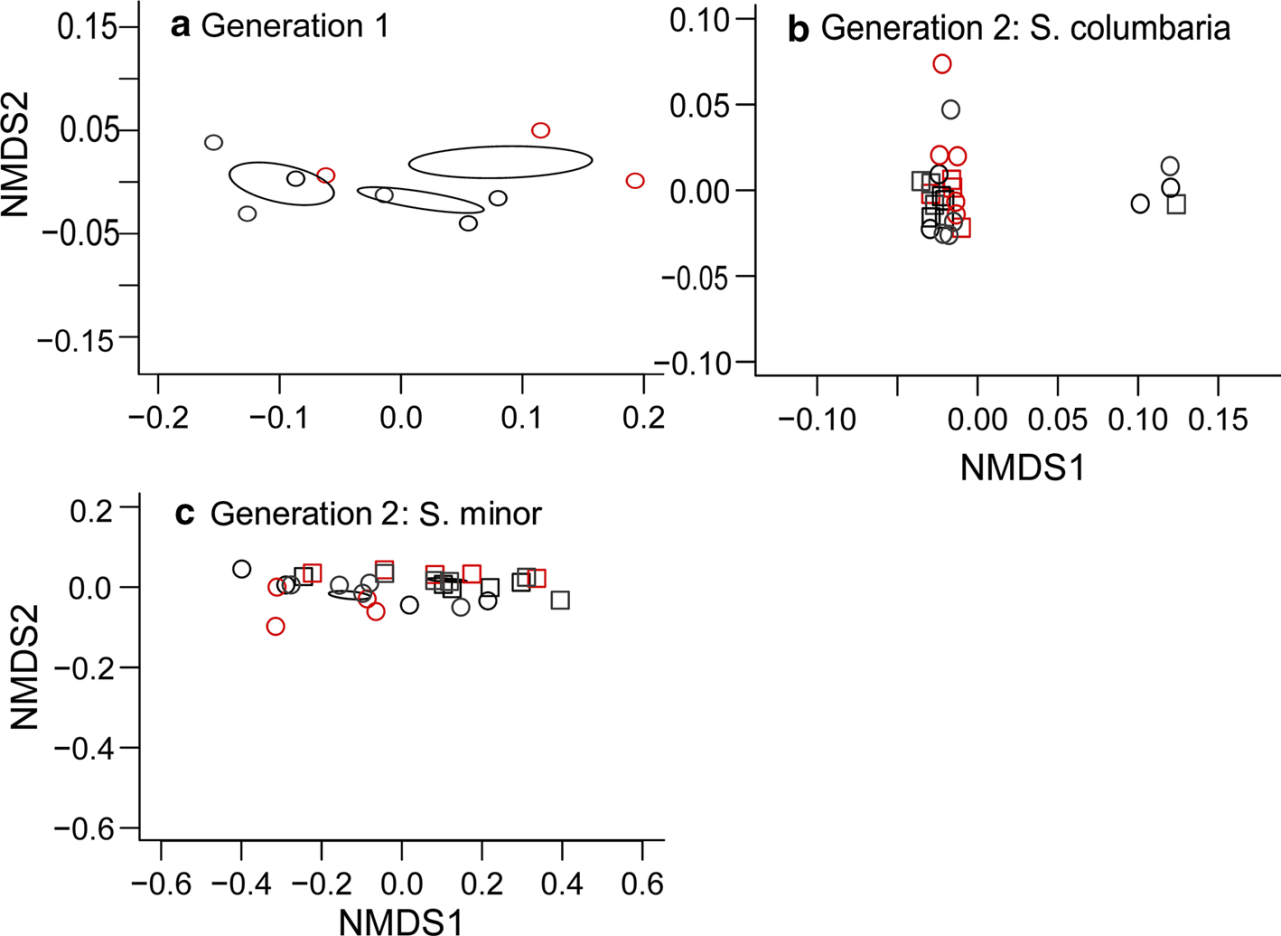
Ordination diagram based on the first two axes of a non-metric multidimensional scaling (NMDS) analysis of the soil microbial community shift after **a** one generation of plants were grown under *Scabiosa columbaria, Sanguisorba minor* or left bare as field soil, **b** the second generation of plants was grown under *S. columbaria*, **c** the second generation of plants was grown under *S. minor*. Black markers: field soil, red: *S. columbaria* conditioning in the first generation, grey: *S. minor* conditioning in the first generation. In plots **b** and **c**, circles are well-watered pots, squares are droughted. The ellipses in panels **a** and **c** denote standard error of the variation between pots. Where they appear, a statistically significant treatment effect was detected. In **a**, there is a significant effect of soil conditioning on the microbial community in the first generation. In **c**, there is a significant effect of the watering treatment on soil microbial community under *S. minor* in the second generation. Please note that a colour version of this figure is available in the online version of this journal

The NMDS analysis of PFLA data revealed that during the first phase of the experiment, the microbial community structures of soil conditioned by either of the plant species were not significantly different from that of the field soil, although communities of the two species were marginally different from one another, albeit in a non-significant way (Fig. 1a; stress: 0.00008, *k* = 2, *R*^2^ = 0.43, *P* = 0.170). Likewise, there were no significant effects of soil conditioning by the two plant species on total PLFA, fungal or bacterial PLFA, the fungal-to-bacterial ratio, or any individual PLFA (Table S1).

### Phase two: drought and plant–soil feedbacks

#### Microbial community

Following the second phase of the experiment, microbial community structure, analysed by NMDS of PLFA data, showed contrasting treatment effects. Soil planted with *S. columbaria* in the feedback phase showed no significant overall shift of microbial community in response to drought or soil conditioning (Fig. 1b; stress: 0.05, *k* = 2, drought: *R*^2^ = 0.01, *P* = 0.56; soil: *R*^2^ = 0.09, *P* = 0.309). Furthermore, ANOVA revealed that there was no effect of drought on total PLFA, fungal or bacterial PLFA, or any individual PFLA (Table S2). There was, however, a weak effect of soil conditioning on total PLFA (*F*_2,20_ = 3.32, *P* = 0.057); unconditioned field soil had slightly lower total PLFA than both conditioned soils. This was primarily driven by the gram negative bacterial biomarker *cis*18:1ω7, which was much lower in field soils (*F*_2,20_ = 4.44, *P* = 0.025). However, the two conditioning treatments did not significantly differ from one another.

For soil planted with *S. minor*, microbial community composition was affected by drought (Fig. 1c; stress: 0.032, *k* = 2, *R*^2^ = 0.39, *P* < 0.001), although no impacts of conditioning were detected (*R*^2^ = 0.04, *P* = 0.66). Total PLFA, a measure of active microbial biomass, was significantly greater in droughted soil (Table S2: *F*_1,22_ = 10.71, *P* = 0.004), as was bacterial PLFA (*F*_1,22_ = 17.58, *P* < 0.001), and gram positive and negative bacteria (*F*_1,22_ = 12.98, *P* = 0.002 and *F*_1,22_ = 19.81, *P* < 0.001, respectively), while the abundance of the fungal PLFA 18:2ω6 was not significantly affected by any of the treatments. The fungal-to-bacterial PLFA ratio was affected by the drought treatment, being lower in soils subjected to drought (*F*_1,22_ = 10.46, *P* = 0.004).

#### Biomass and traits

**Fig. 2:**
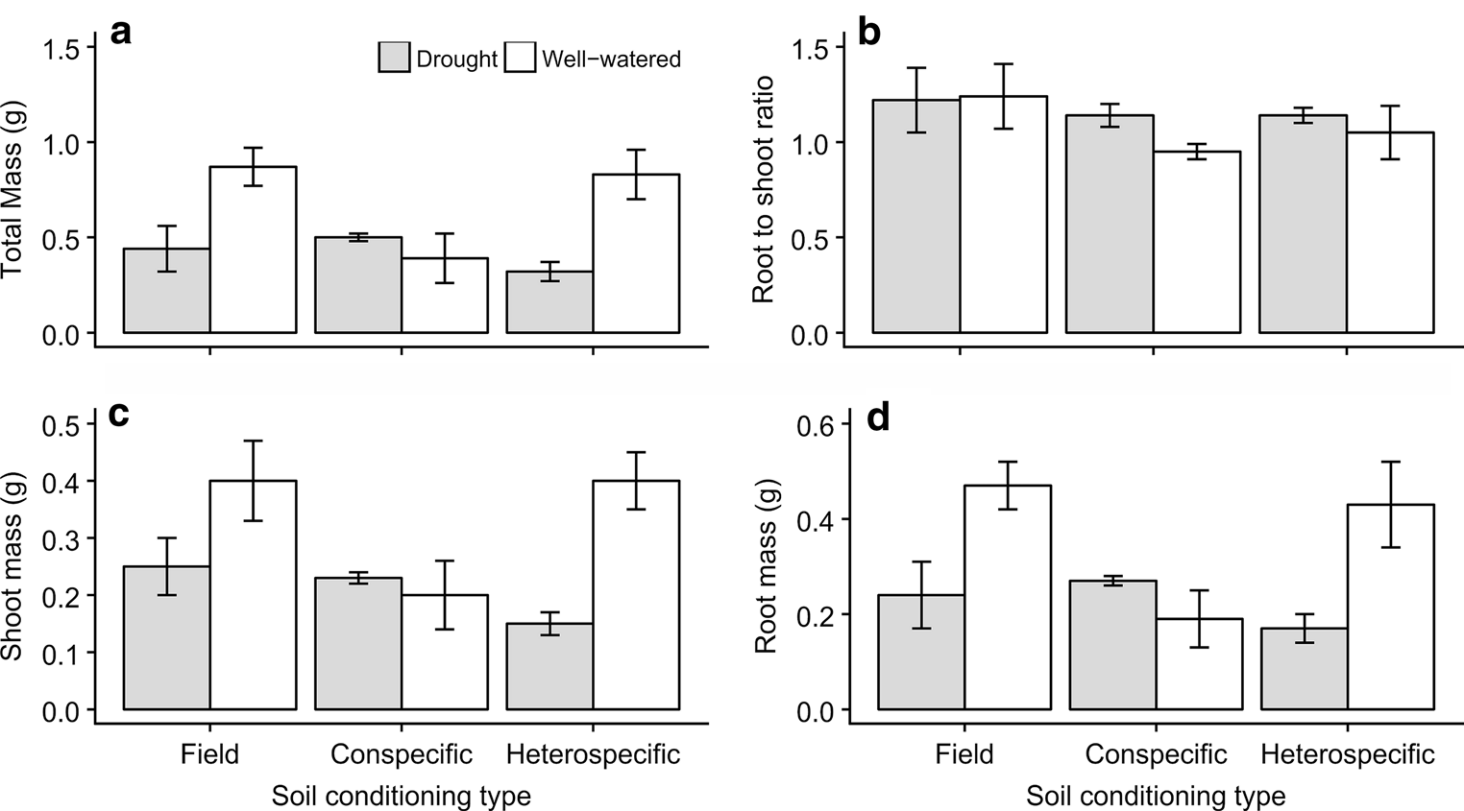
Treatment effects on **a** total biomass, **b** root-to-shoot ratio, **c** shoot biomass and (**d**) root biomass of *Scabiosa columbaria* after one generation of conditioning. Field soils are soils left bare in the first generation, conspecific refers to soils conditioned with *S. columbaria*, heterospecific refers to soils conditioned with *S. minor*. Droughted soils are in grey bars, well-watered soils are in white. Error bars are SEM

The two plant species displayed markedly different responses in terms of root and shoot biomass production, and functional effect traits to soil conditioning and drought. *Scabiosa columbaria* displayed negative PSF for total, shoot and root biomass. In its own soil, the plants were much smaller than in heterospecific soil conditioned by *S. minor* (Table 2; Fig. 2). They were also much smaller in their own soils compared to when grown in unconditioned field soils, although this effect was only apparent under well-watered conditions, leading to a significant interaction between soil-conditioning and drought treatments (total biomass: Fig. 2a; *F*_2,23_ = 4.92, *P* = 0.017, shoots: Fig. 2c; *F*_2,23_ = 4.16, *P* = 0.029, and roots: Fig. 2d; *F*_2,23_ = 4.61, *P* = 0.021; see Table S2 for all statistical output). Under drought, PSFs became neutral, with the biomass of *S. columbaria* being similar in all three soil-conditioning treatments. Also, the root-to-shoot ratio of *S. columbaria* did not change in response to soil conditioning or after drought (Fig. 2b). Collectively, these findings indicate that drought reduced the negative effect of conspecific PSF on this species (Table 2).

**Table 2:** Average plant–soil feedbacks for *S. columbaria* and *S. minor*, calculated using the equation 100(biomass of each conspecific − average biomass of heterospecific)/average biomass of heterospecific (van der Putten et al. 2007)

Conspecific	Heterospecific	Watering treatment	Total biomass	Belowground biomass	Aboveground biomass
*S. columbaria* plant in *S. columbaria* soil	*S. columbaria* plant in *S. minor* soil	Drought	56.90**	56.62**	57.21**
		Well-watered	− 52.81*	− 55.10*	− 50.33*
*S. minor* plant in *S. minor soil*	*S. minor* plant in *S. columbaria* soil	Drought	8.48	12.21	5.82
		Well-watered	25.49*	33.53	19.59

Droughted pots are compared to droughted pots, and well-watered to well-watered. Negative values indicate that the plant is smaller in conspecific soil than heterospecific, while positive values mean the plant is bigger. Significance stars refer to differences from zero using a two-tailed *t* test

**Fig. 3:**
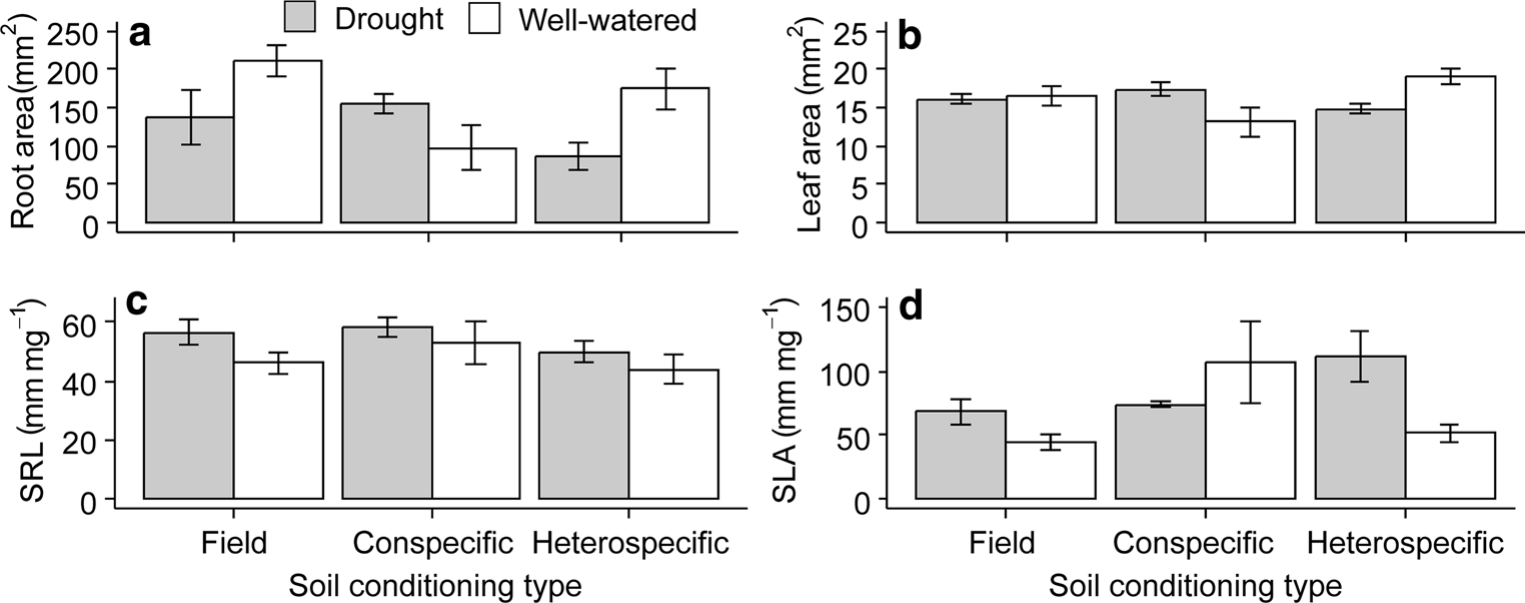
Treatment effects on functional effects traits of *Scabiosa columbaria* after one generation of conditioning: **a** root area, **b** leaf area, **c** specific root length (SRL), **d** specific leaf area (SLA). Field soils are soils left bare in the first generation, conspecific refers to soils conditioned with *S. columbaria*, heterospecific refers to soils conditioned with *S. minor*. Droughted soils are in grey bars, well-watered soils are in white. Error bars are SEM

The negative PSF of *S. columbaria* under well-watered conditions was reflected in a range of plant functional traits. Total root and leaf surface area, and SLA per *S. columbaria* plant were smaller in conspecific soils compared with *S. minor* soils. This trend was reversed when the plants were droughted, and PSF effects on plant traits were positive under drought (roots: Fig. 3a; *F*_2,23_ = 4.50, *P* = 0.023; leaf: Fig. 3b; *F*_2,23_ = 5.99, *P* = 0.008; SLA: Fig. 3d; *F*_2,23_ = 3.66, *P* = 0.043; see Table S2 for all statistical output). The photosynthetic surface area of *S. columbaria* plants grown in unconditioned field soil, by contrast, was not affected by drought, indicating that PSF feedback effects on plant traits of this species were attributable to soil conditioning. By contrast, SRL was not affected by either treatment (Fig. 3c). Root and shoot chemistry of *S. columbaria* was not consistently altered by the PSF treatments, although in both there was higher N in the droughted plants, and field soil had lower shoot N than conditioned soils (Figure S1).

**Fig. 4:**
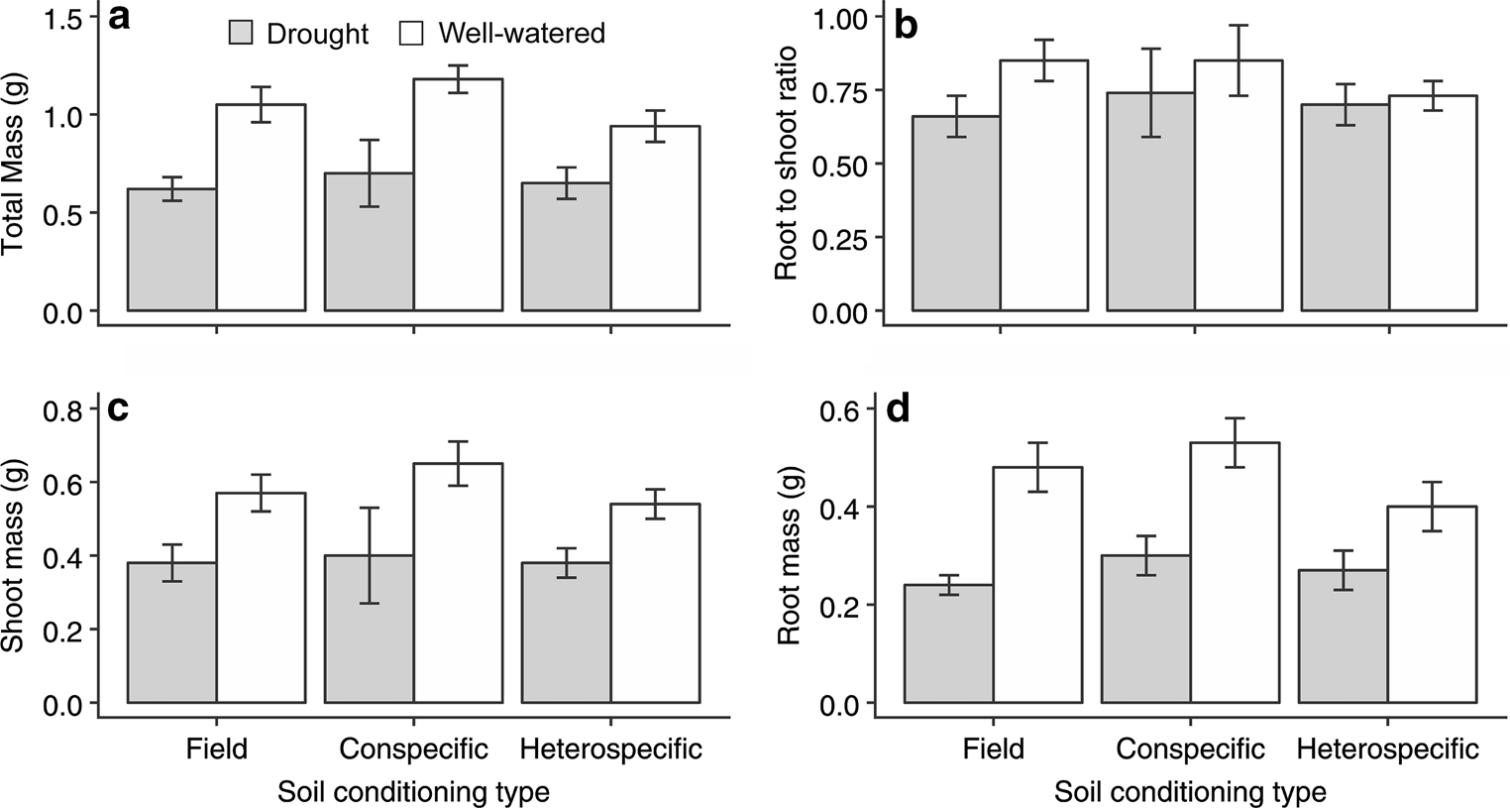
Treatment effects **a** total biomass, **b** root-to-shoot ratio, **c** shoot biomass and **d** root biomass of *Sanguisorba minor* after one generation of conditioning. Field soils are soils left bare in the first generation, conspecific refers to soils conditioned with *S. minor*, heterospecific refers to soils conditioned with *S. columbaria*. Droughted soils are in grey bars, well-watered soils are in white. Error bars are SEM

In contrast to *S. columbaria*, *S. minor* showed positive PSF for total biomass (Table 2), growing larger in conspecific soil than in heterospecific soil conditioned by *S. columbaria*. However, there was no significant effect of PSF on root or shoot biomass. There was a significant effect of drought, where plants were smaller when droughted, but similarly to *S. columbaria*, PSF effects were not apparent under drought (Table 2; Fig. 4; total biomass: *F*_1,24_ = 24.85, *P* < 0.001; root biomass: *F*_1,24_ = 32.86, *P* < 0.001; shoot biomass: *F*_1,24_ = 12.20, *P* = 0.002; see Table S2 for all statistical output). There were no treatment effects on the root-to-shoot ratio (Fig. 4b). Field soil resulted in plants of intermediate size between the two conditioning treatments, but was not significantly different to either.

**Fig. 5:**
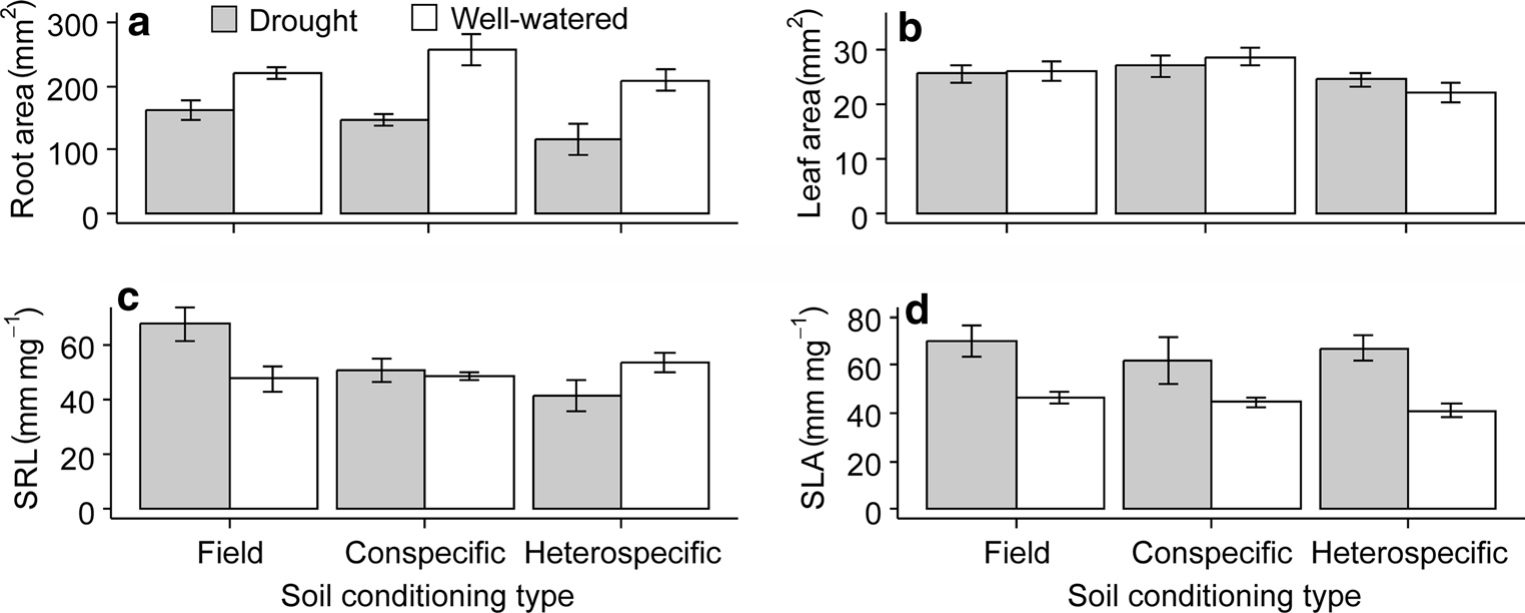
Treatment effects on functional effects traits of *Sanguisorba minor* after one generation of conditioning: **a** root area, **b** leaf area, **c** specific root length (SRL), **d** specific leaf area (SLA). Field soils are soils left bare in the first generation, conspecific refers to soils conditioned with *S. minor*, heterospecific refers to soils conditioned with *S. columbaria*. Droughted soils are in grey bars, well-watered soils are in white. Error bars are SEM

There was no PSF effect on root area of *S. minor*, but droughted plants had smaller root area in all three treatments (Fig. 5a; *F*_1,24_ = 35.96, *P* < 0.001). Leaf area of *S. minor* was greater in conspecific than heterospecific soil (Fig. 5b; *F*_2,24_ = 3.79, *P* = 0.037), but there was no significant effect of drought on this trait. Specific root length (SRL) of *S. minor* did not differ between conspecific and heterospecific soil in well-watered treatments, although plants grown in the field soil had greater SRL under drought than in any of the PSF treatments (Fig. 5c; *F*_2,24_ = 6.19, *P* = 0.007).

Root and shoot N of *S. minor* were not significantly affected by PSF under well-watered conditions. However, under drought, root and shoot N of *S. minor* was significantly higher than in well-watered soils (Figure S1a; *F*_1,24_ = 41.86, *P* < 0.001; Figure A1c; *F*_1,24_ = 35.65, *P* < 0.001, respectively). Additionally, root N of *S. minor* was significantly lower in field soils subject to drought than in either of the droughted conditioned soils (*F*_2,24_ = 4.04, *P* = 0.031). Root and shoot C were not affected by soil conditioning, but they were affected by drought: root C was reduced by drought, whereas shoot C was increased (Figure A1b, *F*_1,24_ = 5.77, *P* = 0.024, Figure A1d; *F*_1,24_ = 6.05, *P* = 0.022, respectively).

### Physiological responses to soil-conditioning and drought treatments

**Fig. 6:**
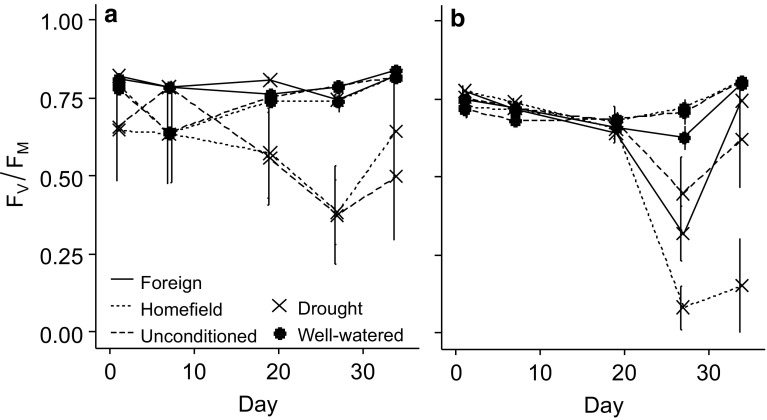
Photosynthetic efficiency (*F*_V_/*F*_M_) of **a** *S. columbaria* and **b** *S. minor* over 5 weeks subjected to soil-conditioning and drought treatments. Filled dots: well-watered; crosses: drought. Solid line: field soil; dashed line: heterospecific soil; dotted line: conspecific soil

The photosynthetic efficiency (*F*_V_/*F*_M_) of *S. columbaria* was not significantly affected by PSF under well-watered conditions. Under drought conditions, however, *F*_V_/*F*_M_ of *S. columbaria* was significantly lower when grown in conspecific than in heterospecific soil, and this effect became stronger the longer the drought went on, with a sharp decline during the last week of the drought (Fig. 6a; soil conditioning: *F*_2,7_ = 23.07, *P* < 0.001; drought: *F*_1,3_ = 11.27, *P* = 0.044). *F*_V_/*F*_M_ of *S. columbaria* recovered to close to pre-drought levels for all treatments following rewetting, and in the final week there were no significant treatment effects.

*F*_V_/*F*_M_ of *S. minor* was also not affected by PSF under well-watered conditions, but it was strongly and negatively affected by drought. At the peak of the drought, *F*_V_/*F*_M_ of *S. minor* was much lower when grown in conspecific relative to heterospecific soil. However, upon rewetting, *F*_V_/*F*_M_ of plants grown in heterospecific soil recovered to control levels, whereas it did not when grown in conspecific soil; as such, the recovery of *F*_V_/*F*_M_ following drought was stalled in conspecific soil. When grown in field soil, *S. minor* also showed a reduction in *F*_V_/*F*_M_, but there was a full recovery upon rewetting (Fig. 6b; drought × soil conditioning: *F*_2,128_ = 7.37, *P* < 0.001) as in heterospecific soil.

#### Treatment effects on soil N

**Fig. 7:**
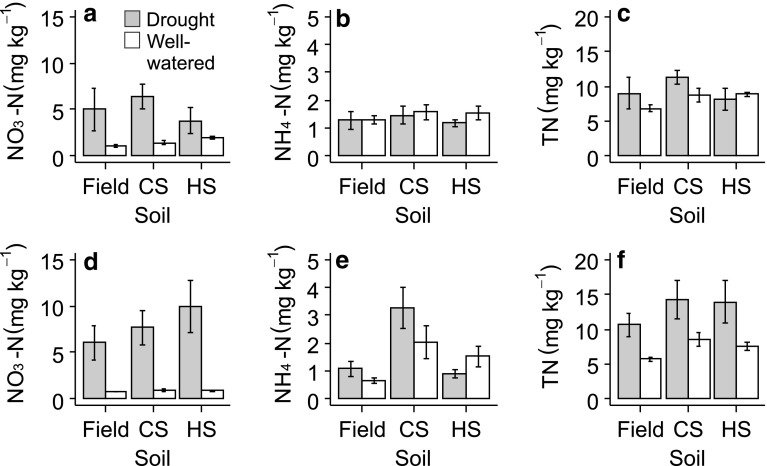
Soil conditioning and drought effects on soil nitrogen forms under *S. columbaria* (**a**–**c**) and *S. minor* (**d**–**f**). Droughted soils are in grey bars, well-watered are in white. *Field* field soil, *CS* conspecific soil, *HS* heterospecific soil. Error bars are SEM

Soil conditioning by *S. columbaria* had no effect on soil concentrations of NO_3_, although drought increased NO_3_ relative to well-watered conditions (Fig. 7a, *F*_1,22_ = 22.10, *P* < 0.001). There was no effect of conditioning with *S. columbaria* or drought on soil NH_4_ concentrations and total soil N. Likewise, soil conditioning by *S. minor* had no effect on NO_3_ concentrations, but NO_3_ was increased by drought compared to well-watered pots (Fig. 7d, *F*_1,24_ = 161.90, *P* < 0.001). However, soil NH_4_ concentrations were significantly higher in soil conditioned by *S. minor* relative to heterospecific soils conditioned by *S. columbaria*, but this measure was not affected by drought (Fig. 7e, *F*_2,24_ = 10.95, *P* < 0.001). Total soil N was not significantly affected by conditioning or drought in soil of *S. minor* (Fig. 7f).

## Discussion

**Fig. 8:**
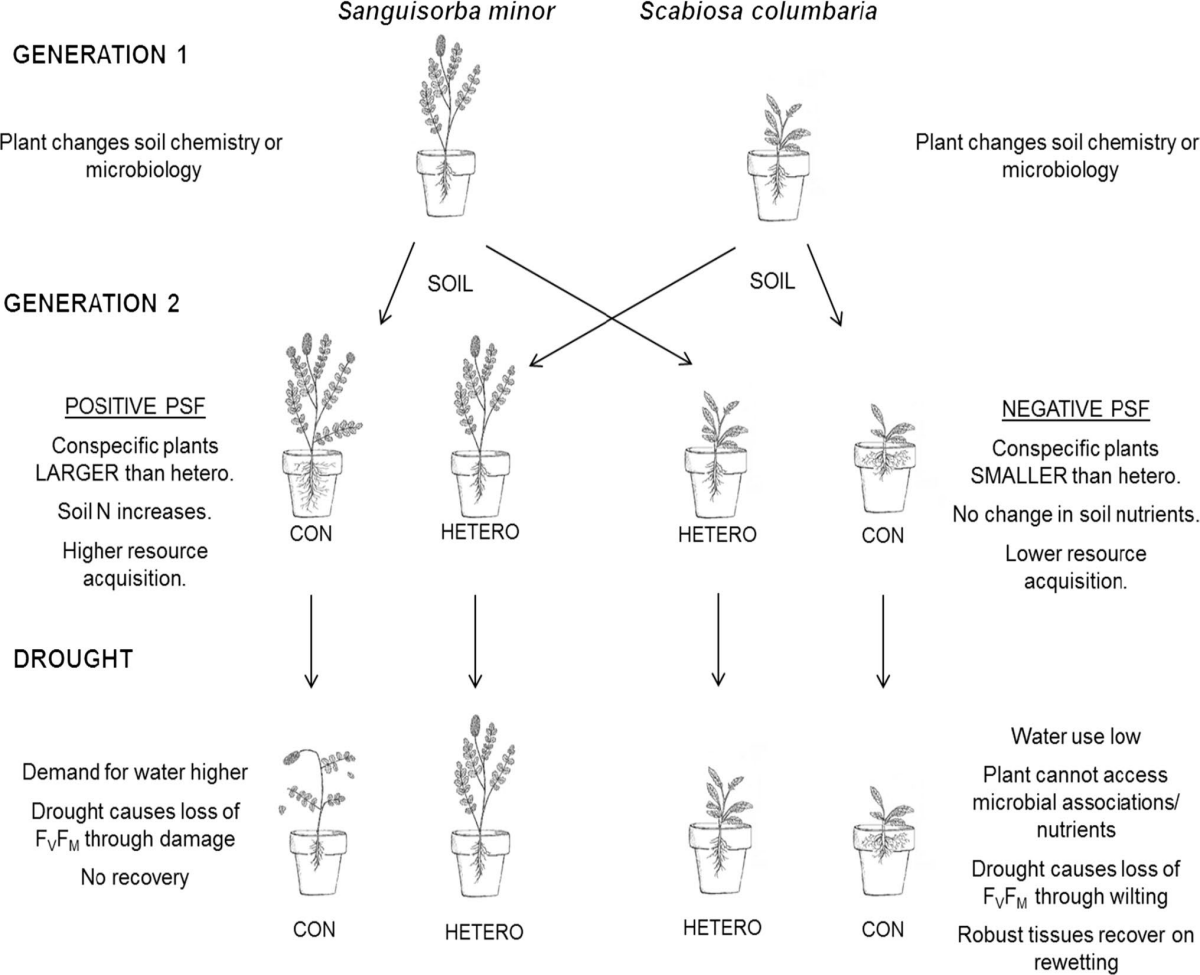
Schematic of results. Soil is conditioned in generation 1 by *Sanguisorba minor* or *Scabiosa columbaria*. In generation 2, the plants are grown in their own (CON) or the other species’ (HETERO) soil. *S. minor* is larger in CON soil, *S. columbaria* smaller relative to when planted in HETERO soil. Under drought, the larger *S. minor* in CON soil loses all photosynthetic efficiency, while the smaller *S. columbaria* in CON soil wilts and recovers

The main objective of this study was to explore how drought alters the outcome of plant–soil feedbacks (PSFs), by altering plant biomass and functional traits of grassland forb species. We also tested whether effects of drought on PSF were related to changes in soil microbial communities and nutrient availability. Finally, we tested whether these responses were related to changes in resilience to drought, which was determined by monitoring photosynthetic efficiency over the course of the drought and after rewetting. A key finding of our study was that drought neutralised plant–soil feedbacks of the forb species that displayed negative feedbacks in conspecific soil; i.e. *S. columbaria*, which resulted in similar biomass and trait values across all conditioning treatments. We found that *S. columbaria* displayed negative feedback under well-watered conditions, as expected from previous studies (Bezemer et al. 2006). But this negative feedback was cancelled out by drought, in that this species performed equally well in conspecific and heterospecific soil in the droughted treatment. In contrast, *S. minor* displayed positive feedback of whole-plant biomass under well-watered conditions in conspecific soil, and this was neutralised by drought, although the droughted plants were generally smaller across all conditioning types (Table 1; Fig. 8). This species also caused an increase in soil NH_4_ in conspecific soil, so the feedback was likely to be driven by abiotic rather than microbial factors. Therefore, the positive feedback increases the requirement for nutrients and, in turn, water in this species. As the two species showed contrasting responses to drought in terms of biomass and functional traits, there were also contrasting effects of drought on photosynthetic efficiency: for *S. columbaria*, which displayed negative feedback under control conditions, we detected rapid recovery of photosynthetic efficiency after rewetting, whereas *S. minor*, which displayed positive feedback, showed virtually no recovery of photosynthetic efficiency after rewetting, which could indicate damage to the leaves and photochemical apparatus (Fig. 8; Maxwell and Johnson 2000). This is likely to be a result of the acquisitive nature of this species under positive feedback.

Feedbacks are the net effect of biotic and abiotic changes in the soil: positive feedback results from a high abundance of symbionts and/or nutrient availability outweighing the negative effect of antagonistic microbes on plant growth, whereas negative feedback is driven by nutrient limitation or an accumulation of pathogens (Bever 2003; van der Putten et al. 2013). As such, it is likely that the neutralising effect of drought on PSFs in our study was due to an altered balance of these factors, possibly through impeding motility through the soil, which resulted in a net biomass and trait change of zero. We do not know the precise mechanisms involved, but for *S. columbaria*, the neutralisation of negative feedback was likely due to drought-induced changes in soil nutrient availability, rather than a shift in microbial community composition. This view is supported by our finding that drought had no detectable effect on microbial community structure of soil planted with this species, as measured by PLFA; but it did significantly increase soil nitrate availability, and hence plant N supply, which could have balanced against negative effects of pathogens on this species in conspecific soil.

In contrast, positive feedback of *S. minor* was likely explained by improved colonisation of roots with arbuscular-mycorrhizal fungi (AMF) in conspecific soil, as suggested by Bezemer et al. (2006), along with high availability of ammonium N, as our results demonstrate. Given that drought had no effect on soil inorganic N availability for this species, it is therefore likely the drought-induced neutralisation of positive feedback was due to a change in soil microbial community structure. In support of this, we observed a significant shift in microbial community composition with drought for this species, measured using PLFA, but no effect of conditioning; it is therefore possible that a drought-induced shift in microbial community under *S. minor* resulted in either loss of beneficial microbes or an increased abundance of pathogenic microbes, which cancelled out positive feedback. This latter suggestion would require higher resolution analysis of microbial community responses to that done here using PLFA, but it is consistent with Kaisermann et al. (2017), who found that drought-induced changes in the performance of the herb *Leontodon hispidus*, which displays positive feedback under non-droughted conditions, were due to a reduction in abundance of beneficial soil microbes and increased abundance of pathogenic microbes.

We also tested how soil conditioning affected the resilience of both species to drought, measured as photosynthetic efficiency. *S. columbaria* showed a high resilience to drought in both heterospecific and conspecific soil; in conspecific soils, droughted plants showed a reduction in photosynthetic efficiency, but then a rapid recovery to near-control levels following rewetting. Recovery means that there was no photochemical damage to the leaves under drought, which is consistent with previous studies showing *S. columbaria* to be drought tolerant (Buckland et al. 1997). Species that show such responses increase solute concentrations in their cells in order to increase osmotic potential during drought, as well as making metabolic adjustments and wilting (Souza et al. 2004). In our study, *S. columbaria* was smaller in droughted heterospecific and field soils, but photosynthetic resilience was high, which could indicate that this species has a range of potential adaptations involving conservative growth and trait expression, in response to water stress. The reduction of photosynthetic efficiency through drought occurs because stomata close to retain water, so CO_2_ cannot be taken up and photosynthesis is down-regulated (Reddy et al. 2004). Excess photons are then lost as thermal energy, which means that they are thermally deactivated, preventing damage to the photosynthetic machinery (Demmig-Adams and Adams 1996). In our study, the physiological limits of this species were not exceeded by drought, as shown by the recovery upon rewetting. Surprisingly, *S. columbaria* grown in unconditioned field soil showed no response to drought, remaining at the same level of photosynthetic efficiency as the well-watered species. There could have been an effect of soil conditioning that lowered water availability compared with in field soils, such as increased soil aggregation (Kaisermann et al. 2017).

As well as having positive PSF, resilience of *S. minor* to drought, measured as recovery of photosynthetic efficiency upon rewetting, was strongly altered by soil conditioning. In particular, when this species was grown in conspecific soil it was associated with very poor resilience to drought, as shown by a decline and lack of recovery in photosynthetic efficiency. Poor photosynthetic resilience of *S. minor* to drought likely occurred partly because this species has a strategy of retaining turgor and putting resources into foraging for water, which was more marked in conspecific soils (Buckland et al. 1997). Bloor and Bardgett (2012) hypothesised that plants that grow in high N soils are more vulnerable to drought because of higher demand for water, which is likely to have exacerbated the stress of the plants in the conspecific soils, which had higher soil N. They also highlighted that increases in turnover rates of plant organs under high N can lower resistance to drought. By growing larger regardless of water availability, *S. minor* therefore reduced structural integrity of leaves and roots (Ryser and Eek 2000). This high growth rate, combined with the lack of available water, resulted in a collapse of photosynthetic efficiency with no recovery. Others have noted that *S. minor* is a drought avoider, and this means that the species maintains high cell water potential and makes no osmotic adjustment, instead relying on stomatal closure (Souza et al. 2004). However, maintaining tissue water content results in drought-induced C starvation injury and metabolic alterations, and possibly hydraulic failure, which is consistent with what we observed in the conspecific treatment (Buckland et al. 1997; McDowell et al. 2008).

One criticism of PSF experiments is that when a greenhouse-based study is employed, soil nutrient depletion can arise to such a degree from the first generation of plant growth, that PSF effects are indistinguishable from the depletion effect (Brinkman et al. 2010). By comparing conditioned soils to field soils that were bare in the first generation, we have shown in every treatment an increase in soil N after soil conditioning. Indeed, the large increase in N observed in the conspecific *S. minor* treatment (but not heterospecific) can be considered as a direct effect of the first generation plant and its associated microbial community. This high N availability was mirrored in the root N content, which was also higher in conspecific than heterospecific or field soils. Therefore, it is unlikely that the plants were suffering any form of nutrient depletion in our experiment. It is also of note that we did not sterilise soils in our study, as commonly done in PSF research (Kardol et al. 2007; Engelkes et al. 2008). Sterilised soils come with their own challenges, not least the flush of nutrients that occurs when the microbial community is killed (van der Putten et al. 2007), and the potential for a wholly artificial microbial community to be acquired from the surroundings (Brinkman et al. 2010). We decided to use field soil that had a pre-existing soil community, so that the conditioning could favour naturally occurring microbial groups instead of incidental arrivals.

## Conclusions

Our results indicate that drought can neutralise the effect of PSF on plant growth. Specifically, we demonstrate that two outwardly similar plant species of calcareous grassland respond differently to PSFs, but under drought these effects are neutralised. Our results show that while positive and negative PSF may disappear under drought, there may be other effects of soil conditioning that affect the species’ ability to recover from stress, and which cannot be encapsulated by measuring biomass or effects traits. We therefore advocate using a suite of measures to disentangle the effects of soil conditioning and drought on physical, morphological and physiological characteristics of the plant species in question.

Calcareous grassland is highly diverse and this is mainly attributed to the poor nutrient availability and low organic matter present in the soils, which prevent dominance of one or a few species. Recent findings by Wagner et al. (2016) indicate that physical disturbance of the sward, creating microsites to allow establishment of specialist species and reducing competition of generalists is crucial for creating species-rich communities in calcareous grassland. While successful establishment is critical, maintaining this species richness in the face of climate stress is frequently overlooked. Here we find that contrasting responses to stress could be one reason that high diversity calcareous grasslands persist in a number of stable states according to abiotic and biotic conditions (Bradshaw 1983; Fagan et al. 2008; Bever et al. 2010). This highlights the need to disentangle effects of soil conditioning on seemingly similar plant species, and to consider them as drivers of many complex responses not limited to biomass effects. It could also offer another explanation for the high species richness seen in calcareous grasslands, because seemingly similar species vary in their responses to plant soil feedback and abiotic stress.

## Electronic supplementary material

Below is the link to the electronic supplementary material.

Supplementary material 1 (DOCX 1003 kb)
