# Supplementary material for: Drought neutralises plant–soil feedback of two mesic grassland forbs
Source: Oecologia. 2018 Feb 5;186(4):1113–25. doi: 10.1007/s00442-018-4082-x (PMC5859691; doi:10.1007/s00442-018-4082-x)

SUPPLEMENTARY MATERIAL

Table S1: Output of ANOVAs testing significance of soil conditioning on soil microbial community in the first generation.

| Response Variable | d.f. | F | p |
| --- | --- | --- | --- |
| Total PLFA | 2,6 | 2.24 | 0.188 |
| Total bacteria | 2,6 | 2.27 | 0.185 |
| Total fungi | 2,6 | 2.16 | 0.197 |
| Fungal to bacterial ratio | 2,6 | 0.41 | 0.680 |
| Gram positive bacteria | 2,6 | 1.98 | 0.218 |
| Gram negative bacteria | 2,6 | 2.47 | 0.165 |

Table S2: Output of ANOVAs testing significance of soil conditioning on biomass, plant traits and soil properties in the second generation. Highlighted text denotes significance at the p<0.05 level.

1. *Scabiosa columbaria*

|  | | Root mass | | | Leaf mass | | Total biomass | | | Root to shoot ratio | |
| --- | --- | --- | --- | --- | --- | --- | --- | --- | --- | --- | --- |
|  |  | |  |  |  |  | |  |  |  |  |
| Factor | d.f. | | F | p | F | p | | F | p | F | p |
| Water | 1 | | **8.50** | **0.010** | **9.20** | **0.010** | | **10.72** | **0.000** | 0.75 | 0.397 |
| Soil | 2 | | 2.6 | 0.096 | 2.86 | 0.079 | | 2.45 | 0.109 | 1.41 | 0.265 |
| Interaction | 2 | | **4.60** | **0.020** | **4.20** | **0.030** | | **4.92** | **0.020** | 0.38 | 0.692 |
|  | 23 | |  |  |  |  | |  |  |  |  |
|  |  | |  |  |  |  | |  |  |  |  |
|  |  | Root area | | | SRL | | Leaf area | | | SLA | |
|  |  | |  |  |  |  | |  |  |  |  |
| Factor | d.f. | | F | p | F | p | | F | p | F | p |
| Water | 1 | | 2.98 | 0.098 | 3.13 | 0.090 | | 0.06 | 0.805 | 1.76 | 0.198 |
| Soil | 2 | | 2.32 | 0.121 | 1.65 | 0.214 | | 1.32 | 0.289 | 2.34 | 0.120 |
| Interaction | 2 | | **4.50** | **0.020** | 0.16 | 0.849 | | **5.99** | **0.010** | **3.66** | **0.043** |
|  | 23 | |  |  |  |  | |  |  |  |  |

1. *Sanguisorba minor*

|  |  | Root mass | | Leaf mass | | Total biomass | | Root to shoot ratio | |
| --- | --- | --- | --- | --- | --- | --- | --- | --- | --- |
|  |  |  |  |  |  |  |  |  |  |
| Factor | d.f. | F | p | F | p | F | p | F | p |
| Water | 1 | **32.86** | **<0.001** | **12.20** | **0.002** | **24.85** | **<0.001** | 0.23 | 0.635 |
| Soil | 2 | 2.00 | 0.158 | 0.46 | 0.636 | 1.20 | 0.318 | 1.29 | 0.295 |
| Interaction | 2 | 1.01 | 0.381 | 0.19 | 0.826 | 0.47 | 0.632 | 0.94 | 0.406 |
|  | 24 |  |  |  |  |  |  |  |  |
|  |  |  |  |  |  |  |  |  |  |
|  |  | Root area | | SRL | | Leaf area | | SLA | |
|  |  |  |  |  |  |  |  |  |  |
| Factor | d.f. | F | p | F | p | F | p | F | p |
| Water | 1 | **35.96** | **<0.001** | 0.84 | 0.370 | 0.00 | 0.977 | 3.11 | 0.091 |
| Soil | 2 | 2.50 | 0.103 | 2.70 | 0.087 | **3.79** | **0.037** | 0.86 | 0.437 |
| Interaction | 2 | 1.15 | 0.335 | **6.19** | **0.007** | 0.77 | 0.473 | 0.82 | 0.453 |
|  | 24 |  |  |  |  |  |  |  |  |

Figure S1: Treatment effects on tissue chemistry in both species.

1. *Scabiosa columbaria* treatment effects on tissue chemistry. Significance stars are as follows: *=p<0.05, **=p<0.01, ***=p<0.001.


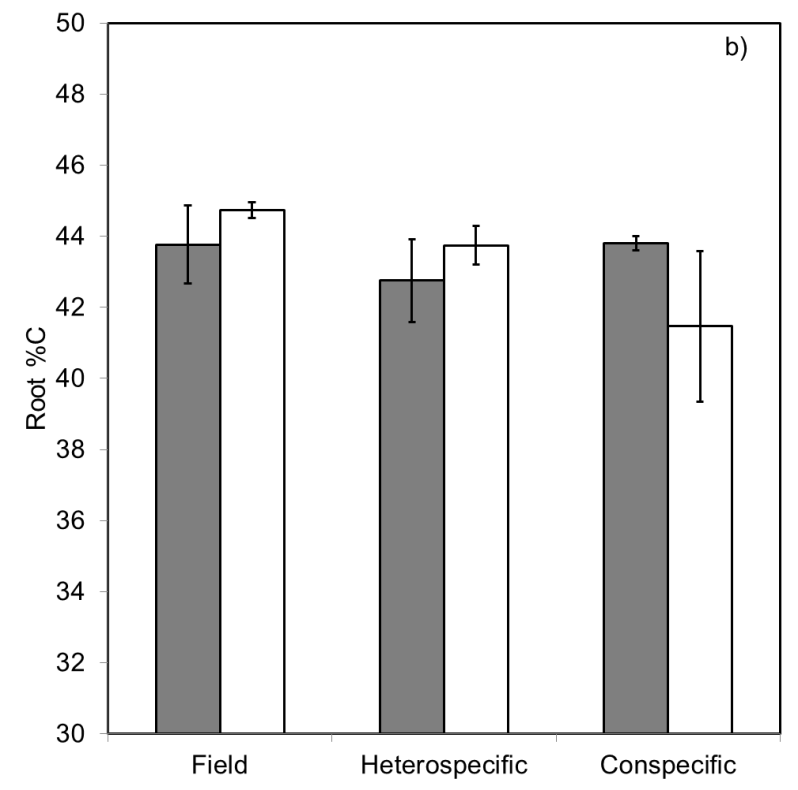

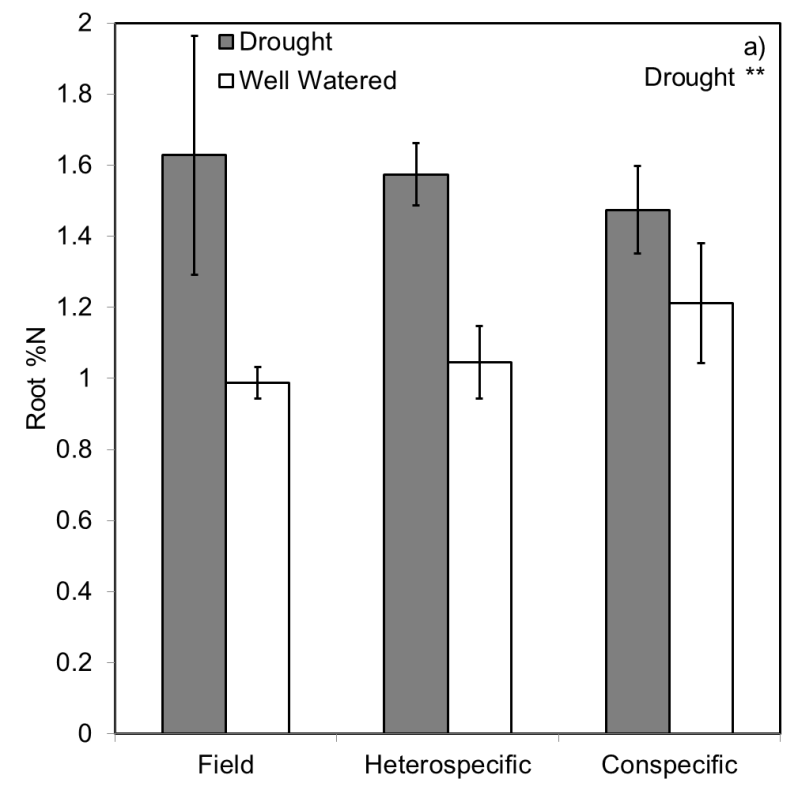

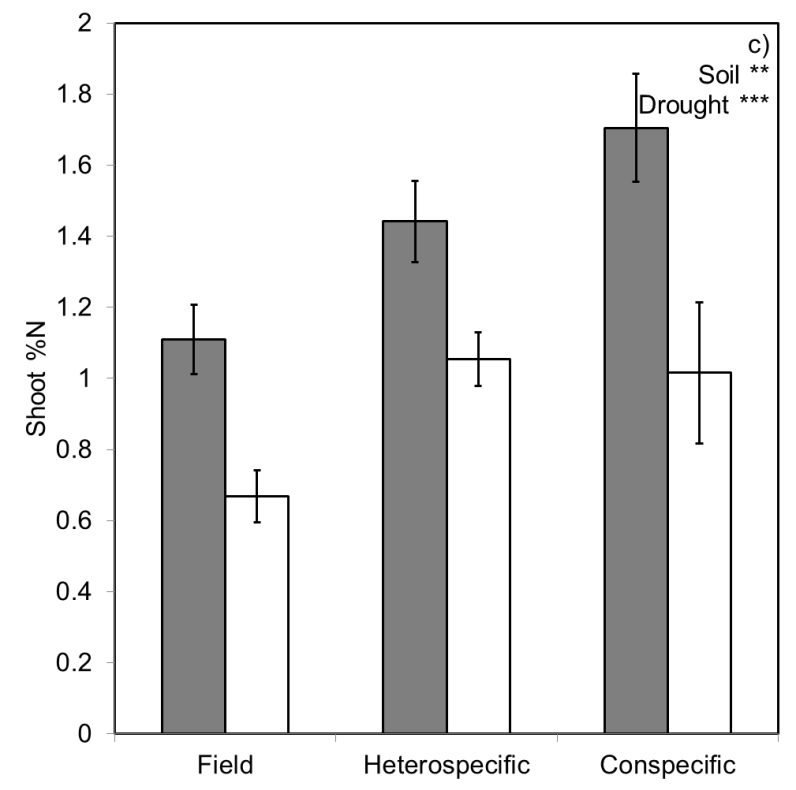

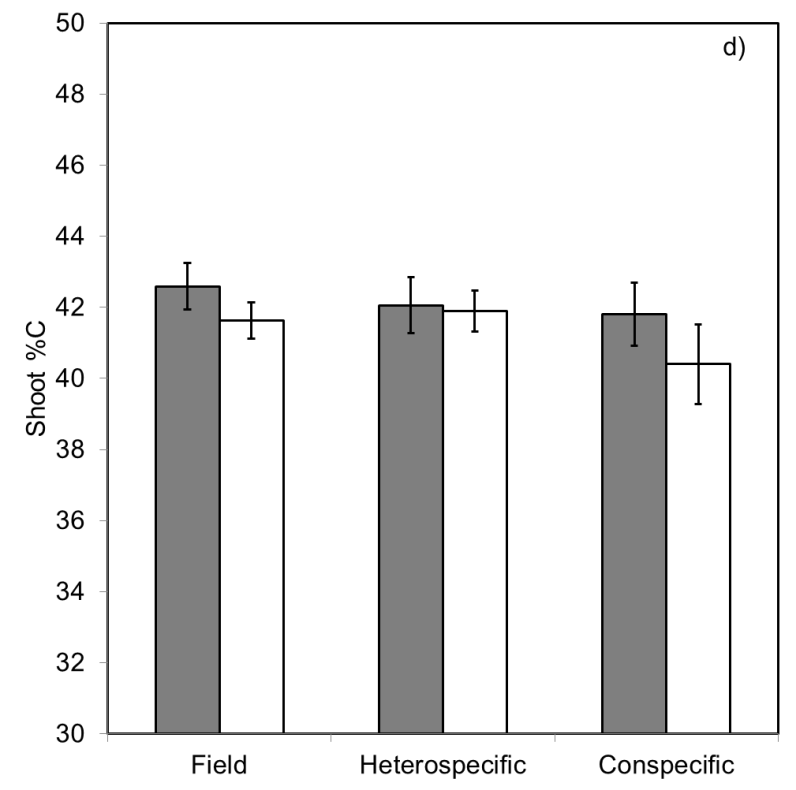


1. *Sanguisorba minor*. Significance stars are as follows: *=p<0.05, **=p<0.01, ***=p<0.001.


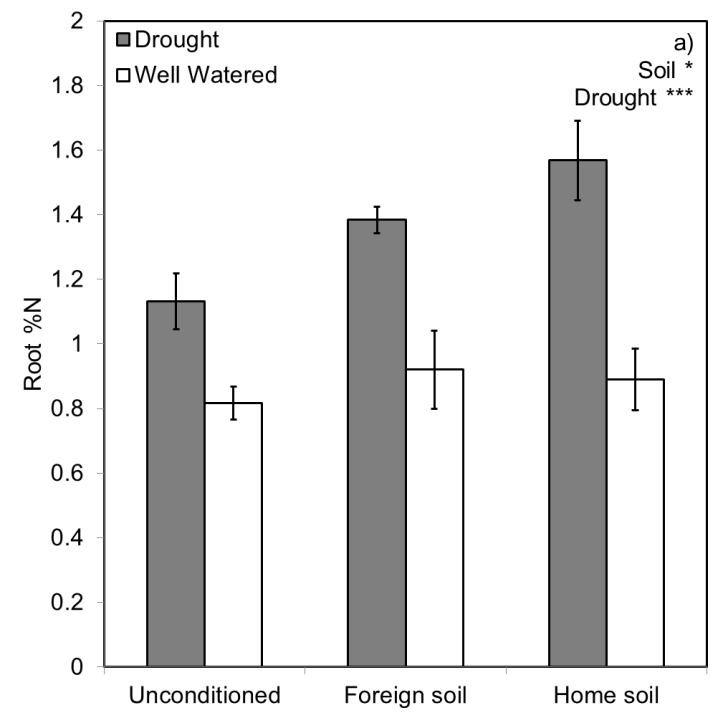

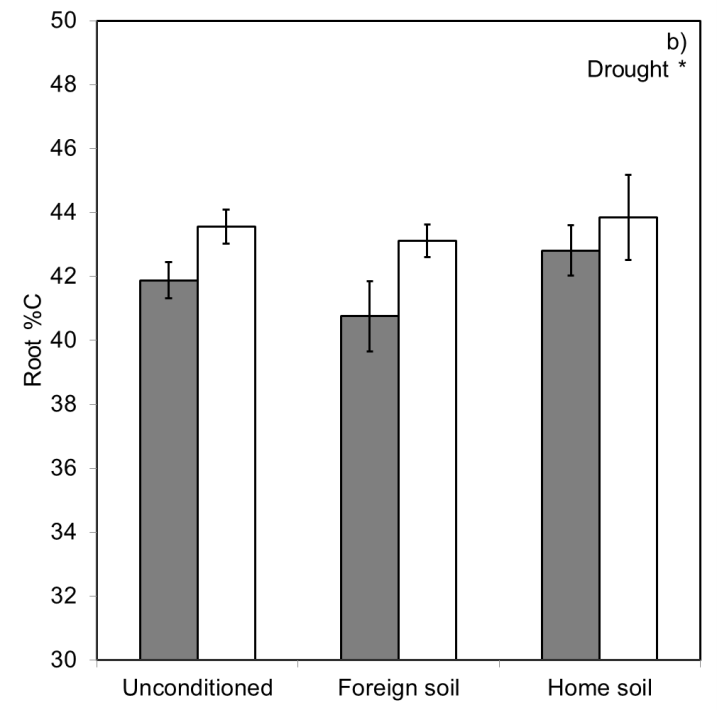

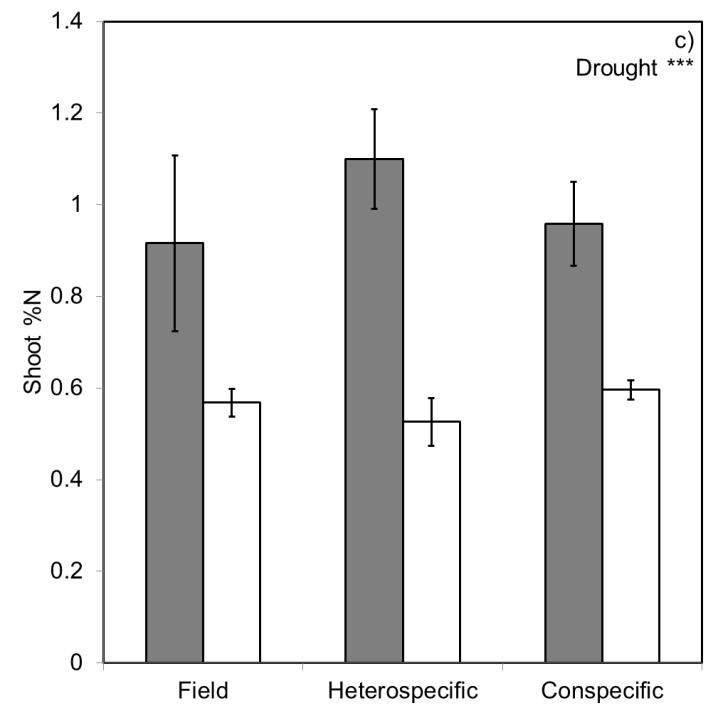

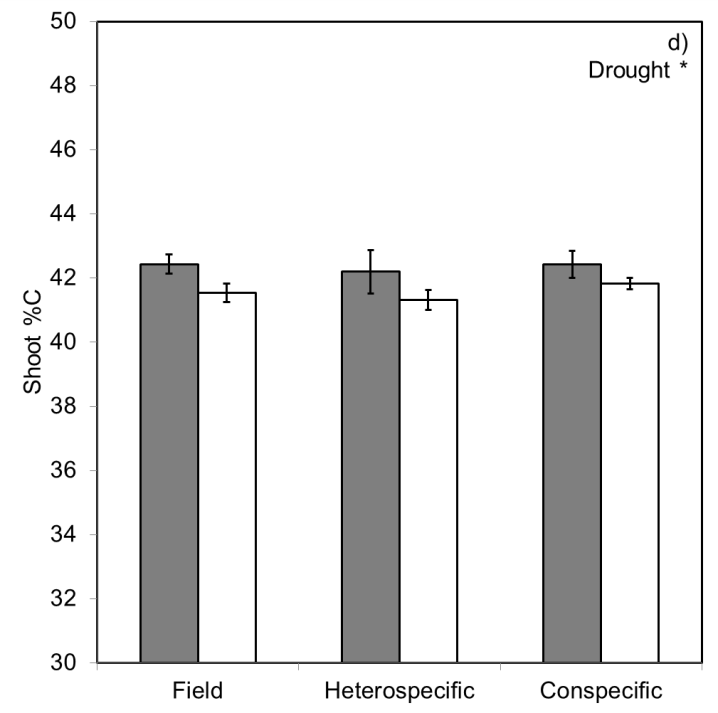


Figure S2: Correlations between biomass and photosynthetic efficiency of a) *S. columbaria* and b) *S. minor*.


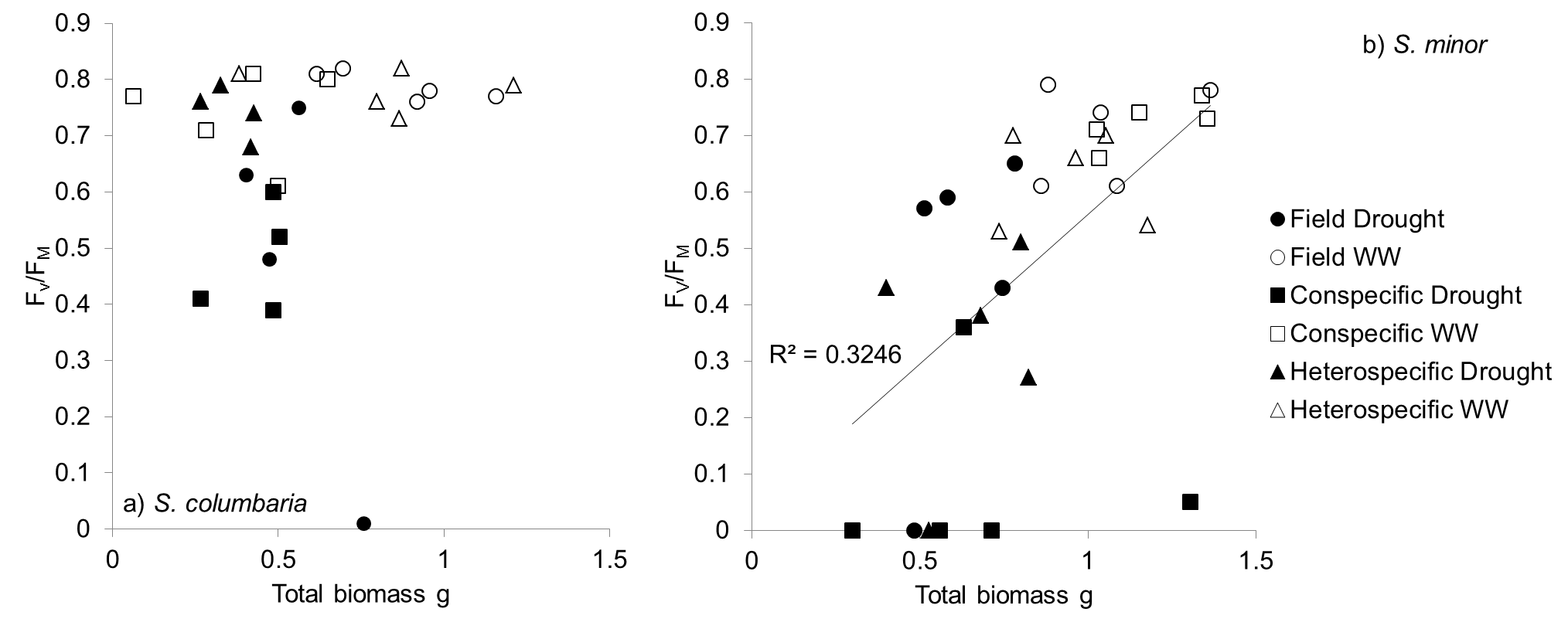

Supplement: Supplementary file 1 — Supplementary material 1 (DOCX 1003 kb) [file 442_2018_4082_MOESM1_ESM.docx]
